# Detection of *Leptospira kirschneri* in a short-beaked common dolphin (*Delphinus delphis delphis*) stranded off the coast of southern California, USA

**DOI:** 10.1186/s12917-024-04111-x

**Published:** 2024-06-20

**Authors:** K. C. Prager, Kerri Danil, Elyse Wurster, Kathleen M. Colegrove, Renee Galloway, Niesa Kettler, Rinosh Mani, Ryelan F. McDonough, Jason W. Sahl, Nathan E. Stone, David M. Wagner, James O. Lloyd-Smith

**Affiliations:** 1 Department of Ecology and Evolutionary Biology, University of California Los Angeles, Los Angeles, CA 90095 USA; 2 Southwest Fisheries Science Center, National Marine Fisheries Service, NOAA, La Jolla, CA 92037 USA; 3 Ocean Associates Inc. Under Contract to Southwest Fisheries Science Center, National Marine Fisheries Service, NOAA, La Jolla, CA 92037 USA; 4 Zoological Pathology Program, University of Illinois College of Veterinary Medicine, 3300 Golf Rd, Brookfield, IL 60513 USA; 5 National Center for Emerging and Zoonotic Infectious Diseases, Centers for Diseases Control and Prevention, Atlanta, GA 30333 USA; 6 Veterinary Diagnostic Laboratory, College of Veterinary Medicine, Michigan State University, East Lansing, Michigan, 48825 USA; 7 The Pathogen and Microbiome Institute, Northern Arizona University, Flagstaff, AZ 86011 USA

**Keywords:** *Leptospira*, Cetacean, Marine mammal, Common dolphin, *Delphinus delphis*, Northeastern Pacific

## Abstract

**Background:**

Pathogenic *Leptospira* species are globally important zoonotic pathogens capable of infecting a wide range of host species. In marine mammals, reports of *Leptospira* have predominantly been in pinnipeds, with isolated reports of infections in cetaceans.

**Case presentation:**

On 28 June 2021, a 150.5 cm long female, short-beaked common dolphin (*Delphinus delphis delphis*) stranded alive on the coast of southern California and subsequently died. Gross necropsy revealed multifocal cortical pallor within the reniculi of the kidney, and lymphoplasmacytic tubulointerstitial nephritis was observed histologically. Immunohistochemistry confirmed *Leptospira* infection, and PCR followed by *lfb1* gene amplicon sequencing suggested that the infecting organism was *L.kirschneri. Leptospira* DNA capture and enrichment allowed for whole-genome sequencing to be conducted. Phylogenetic analyses confirmed the causative agent was a previously undescribed, divergent lineage of *L.kirschneri*.

**Conclusions:**

We report the first detection of pathogenic *Leptospira* in a short-beaked common dolphin, and the first detection in any cetacean in the northeastern Pacific Ocean. Renal lesions were consistent with leptospirosis in other host species, including marine mammals, and were the most significant lesions detected overall, suggesting leptospirosis as the likely cause of death. We identified the cause of the infection as *L.kirschneri*, a species detected only once before in a marine mammal – a northern elephant seal (*Mirounga angustirostris*) of the northeastern Pacific. These findings raise questions about the mechanism of transmission, given the obligate marine lifestyle of cetaceans (in contrast to pinnipeds, which spend time on land) and the commonly accepted view that *Leptospira* are quickly killed by salt water. They also raise important questions regarding the source of infection, and whether it arose from transmission among marine mammals or from terrestrial-to-marine spillover. Moving forward, surveillance and sampling must be expanded to better understand the extent to which *Leptospira* infections occur in the marine ecosystem and possible epidemiological linkages between and among marine and terrestrial host species. Generating *Leptospira* genomes from different host species will yield crucial information about possible transmission links, and our study highlights the power of new techniques such as DNA enrichment to illuminate the complex ecology of this important zoonotic pathogen.

## Background/introduction

Leptospirosis, the disease caused by infection with pathogenic species within the genus *Leptospira*, is a globally important zoonosis [1]. There are 41 pathogenic *Leptospira* species and hundreds of known pathogenic serovars, each with slightly different characteristics, host affinity and host–pathogen interactions [1–8]. Broadly, all mammals are believed to be susceptible to *Leptospira* infection, and infection has also been detected by culture, hamster inoculation, and/or polymerase chain reaction (PCR) in a range of frogs, snakes, and turtles [4, 9–16]. The full range of host–pathogen interactions for *Leptospira* is still being uncovered, with significant challenges arising from the diversity of pathogenic *Leptospira* strains, logistic hurdles in collecting samples from possible hosts, weaknesses in available tools for diagnosis and strain identification, and the broad spectrum of clinical presentations that accompany infections.

Knowledge of infection and transmission of *Leptospira* is based mostly on studies of terrestrial mammals. In mammalian hosts, infections can be completely subclinical, or can present with clinical signs ranging from flu-like symptoms (fever, muscle aches, headache) to pulmonary manifestations, reproductive failure, liver or renal failure, and even death [1, 2, 4, 17]. In infected hosts, leptospires ultimately colonize the kidneys and then are shed in urine; shedding can continue for months to years in some individuals or host species [4]. Leptospires may also colonize other sites, including reproductive tissues [4]. Clinical disease in infected hosts is due to the damage caused by leptospires during initial systemic infection and eventual tissue colonization, as well as the host inflammatory response against the pathogen [1]. Gross and histopathologic lesions detected in cases of leptospirosis can vary depending on host and infecting species, and reflect the bacterial virulence and host immune responses [4]. However, renal lesions are frequent and are characterized by tubulointerstitial nephritis or glomerulonephritis [2, 4, 18, 19]. The most common routes of transmission are direct contact with urine or indirect contact with urine-contaminated soil or water; intact skin is a strong barrier to infection, but damaged skin and mucous membranes are important routes of infection [20]. Vertical transmission can occur, and contact with infectious aborted tissues or sexual contact can also lead to transmission [4]. Leptospire survival in the environment likely varies by *Leptospira* species and genotype, as well as by environmental conditions, with survival ranging from hours to as long as months, and in some cases over a year [8, 21–23]. Some of the shortest reported survival times were recorded for seawater [23] and salts may be inhibitory for pathogenic *Leptospira* in the absence of nutrients [24].

In marine mammals, reports of *Leptospira* have predominantly been noted in pinnipeds, with isolated reports of infections in cetaceans. *Leptospira interrogans* serovar Pomona has been circulating endemically in California sea lions (*Zalophus californianus*), with seasonal outbreaks occurring yearly since at least 1984 [25–30]. *Leptospira* in other pinnipeds has also been documented, especially in the eastern Pacific Ocean, with seropositivity or infection reported in northern fur seals (*Callorhinus ursinus*), northern elephant seals (*Mirounga angustirostris*), Pacific harbor seals (*Phoca vitulina richardsi*), and Steller sea lions (*Eumetopias jubatus*) on the west coast of the United States (northeastern Pacific Ocean); South American sea lions (*Otaria byronia*) along the coast of Chile; and manatees (*Trichechus inunguis*) in the Peruvian Amazon [25–27, 31–39]. In contrast, reports of *Leptospira* infection in cetaceans are quite rare, and *Leptospira* infection or seropositivity has not been previously detected in a cetacean host in the northeastern Pacific Ocean.

Globally, only eight cetacean species have been shown definitively to be infected with *Leptospira*. Two isolates with 98 and 99% similarity, respectively, to *L.interrogans* serovar Copenhageni were cultured from kidneys of a Fraser’s dolphin (*Lagenodelphis hosei*) and a melon-headed whale (*Peponocephala electra*) from the Philippines in 2017 [40]. An isolate that is suspected to be *L. interrogans* based on sequence results was cultured from the kidney of a newborn southern right whale (*Eubalaena australis*) that stranded dead in Argentina in 2010 [41]. An isolate of *L. interrogans* serovar Pomona was cultured from a common bottlenose dolphin (*Tursiops truncatus*) that stranded along the coast of Sardinia, Italy in 2016 [42]. Torres et al. [43] detected *Leptospira* DNA using *LipL*32-PCR in a Clymene dolphin (*Stenella clymene*), 10 Guiana dolphins (*Sotalia guianensis*), seven La Plata dolphins (*Pontoporia blainvillei*), one rough-toothed dolphin (*Stenobredanensis*), and a common bottlenose dolphin. Genetic characterization using *secY* gene sequences of *Leptospira* detected in three of these PCR positive animals—one each of a Clymene, a Guiana, and a La Plata dolphin—identified them as *L. interrogans* with an identity > 99%, and serological classification indicated > 99% similarity with the Icterohaemorrhagiae serogroup. Serologic evidence of *Leptospira* infection (current or historic) and/or detection using a PCR primer that targets both pathogenic and non-pathogenic *Leptospira spp.* has been reported in an additional nine cetaceans [40].

Here we add to the limited body of knowledge regarding *Leptospira* in cetaceans. We report the first detection of *Leptospira* in a short-beaked common dolphin (*Delphinus delphis delphis*), and the first detection in any cetacean in the northeastern Pacific Ocean. We identify the species as *L.kirschneri* and show that infection was associated with tubulointerstitial nephritis, which was the most significant lesion observed in this animal and the likely cause of death. Given the extremely limited body of knowledge regarding *Leptospira* in cetaceans and the marine ecosystem in general, this provides valuable new data on host range. This report raises pertinent questions about the ecology of *Leptospira* in the marine environment, including how the pathogen may transmit between cetaceans and whether these obligate marine hosts play a more significant role in *Leptospira* circulation than is currently recognized.

## Case presentation

**Fig. 1:**
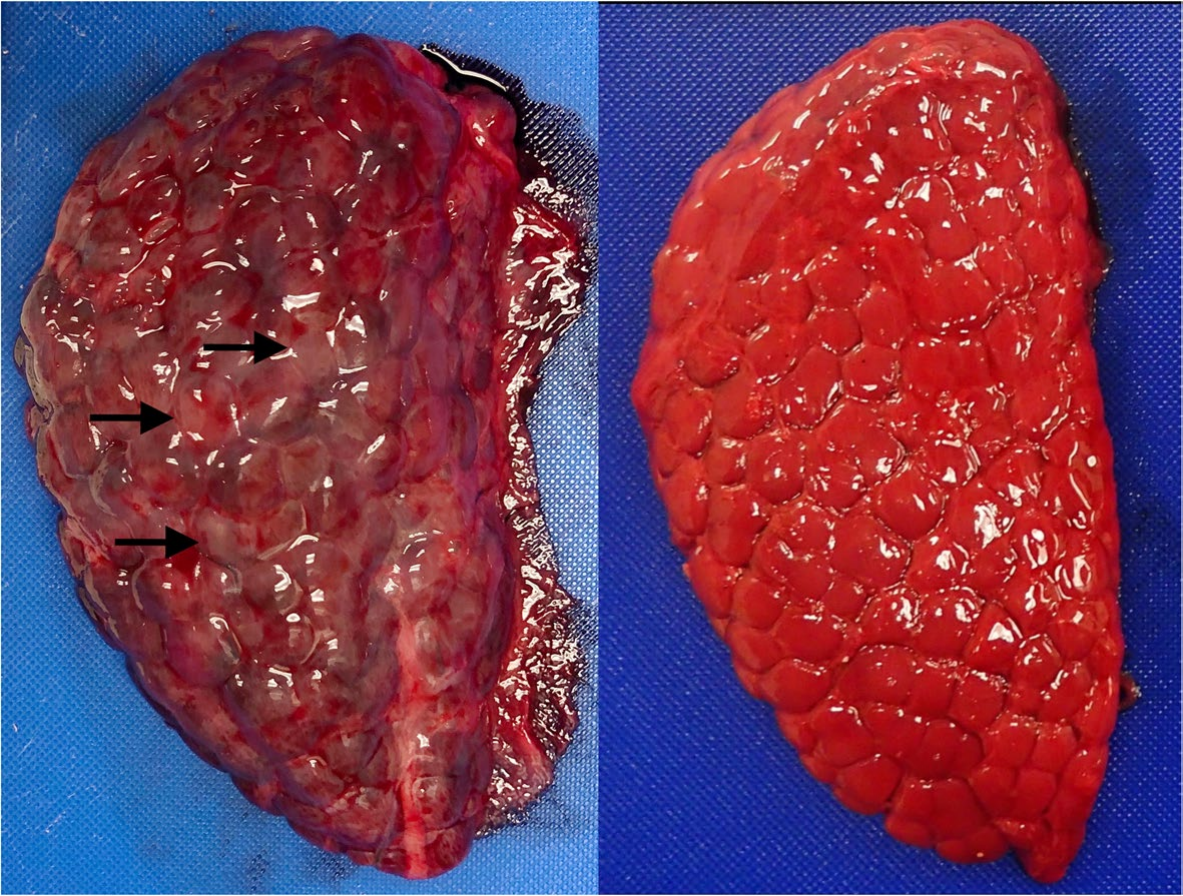
Kidneys of short-beaked common dolphins. The kidney from BLH0012, infected with *Leptospira*, is on the left and has multifocal cortical pallor (indicated by black arrows). On the right is a ‘normal’ kidney from a dolphin

On 28 June 2021, a 150.5 cm long female, short-beaked common dolphin (BLH0012) stranded alive along the coast of southern California (Ponto Beach, Carlsbad, San Diego County) and subsequently died. The carcass was refrigerated at 4͒°C until necropsied on 30 June 2021. Gross necropsy observations included normal body condition [44], an empty stomach, pale yellow intestinal contents, and multifocal cortical pallor within the reniculi of the kidney (Fig. 1). All other organs appeared normal on the gross exam. A standard set of tissue samples were collected and placed in 10% neutral buffered formalin, processed routinely for paraffin embedding, sectioned at 5 μm, stained with hematoxylin and eosin (H&E), and examined microscopically. The age of this dolphin was estimated to be two years old via counts of growth layers in its teeth, using methods outlined in Danil and Chivers [45]. Based on necropsy observations and estimated age, this individual was not sexually mature and was likely still nursing.

**Fig. 2:**
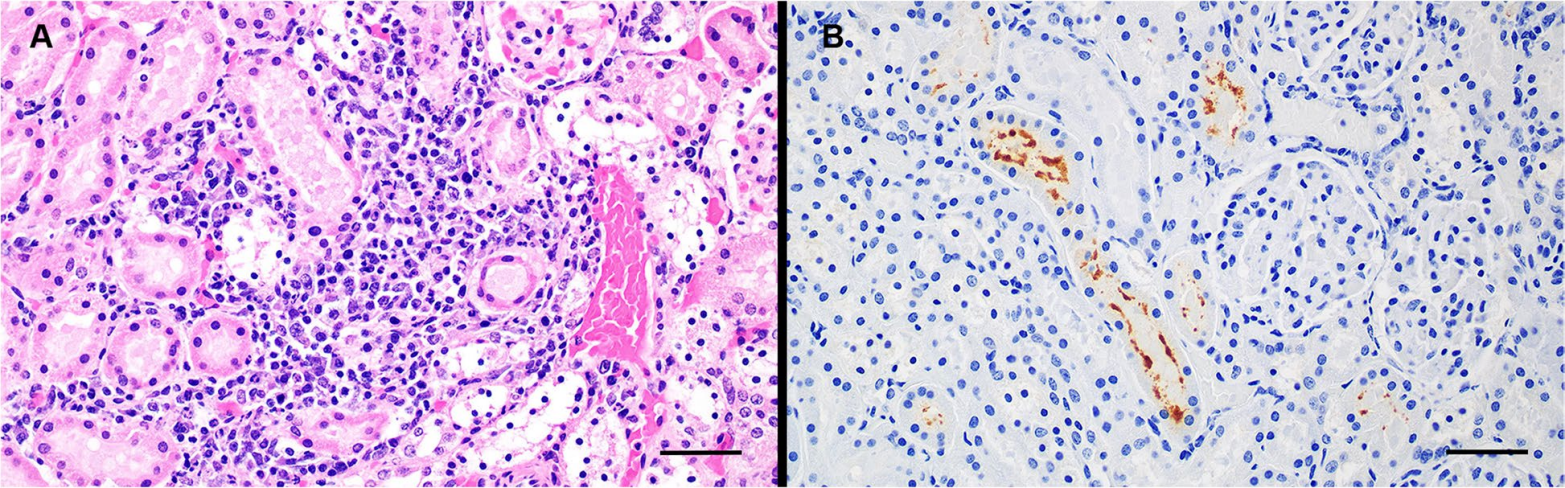
Photomicrographs of kidney from short-beaked common dolphin (BLH0012) with leptospirosis. **A** Hematoxylin and eosin stained section with lymphoplasmacytic tubulointerstitial nephritis. Bar = 20 μm. **B** Kidney stained with a polyclonal antibody directed against *Leptospira* sp. There is dark brown positive staining of material within tubules. Bar = 20 μm

Histologically there was moderate lymphoplasmacytic tubulointerstitial nephritis. Multifocally, renal cortical tubules were surrounded and occasionally disrupted by moderate numbers of plasma cells and fewer lymphocytes. Some affected tubules were dilated, had attenuated epithelium, and contained pale eosinophilic granular material (Fig. 2A). No glomerular lesions were apparent. Other lesions observed in the animal were consistent with debilitation and recent inanition. Immunohistochemistry (IHC) of paraffin-embedded kidney sections was performed using a streptavidin–biotin method and a *Leptospira*-specific polyclonal antibody (National Veterinary Services Laboratory, Ames, Iowa, USA) directed against *L. interrogans* serovars Bratislava, Canicola, Copenhageni (Icterohaemorrhagiae), Hardjo, and Pomona, and *L.kirschneri* serovar Grippotyphosa [33]. Multiple renal tubules, both near and distant from areas of inflammation contained wispy, IHC-positive antigenic staining in the tubular lumens (Fig. 2B). No antigen was demonstrated in negative control sections.

We conducted real-time PCR analysis on fresh frozen kidney to detect and differentiate *L. interrogans, L.kirschneri, L. borgpetersenii* and *L.noguchii*, as described by Ferreira et al. [46] (2014), with the following modification: PerfeCTa Tough Mix Low Rox master mix (Quantabio) and appropriate thermocycling conditions for this master mix were used. We also conducted *Leptospira* genotyping PCR using primers targeting the *lfb1* gene, followed by amplicon sequencing [47]. These methods indicated that the infecting organism most closely resembled *L.kirschneri*. In an effort to determine the specific *Leptospira* serovar and strain, dolphin kidney was cultured for growth of *Leptospira* spp. In brief, tissue was macerated using a Micro-Biomaster Stomacher-80 (Seward Inc., Port St Lucie, FL) with 10 mL of 7.2 pH phosphate buffered saline. The homogenate was filtered through a 0.4 µm filter. The filtrate was inoculated into the *Leptospira* semi-solid (modified EMJH) growth media [48]. A 1:10 dilution of the filtrate in liquid *Leptospira* media (modified EMJH) was also inoculated in the *Leptospira* semi-solid media. The cultures were incubated at 28–29 degrees C for 2 months. Cultures were visually inspected weekly for growth/Dinger zone, but no growth was observed.

To enable genomic level species identification and phylogenetic analyses, DNA extracted from the *Leptospira* positive kidney sample (BLH0012) was subjected to pan pathogenic *Leptospira* DNA capture and enrichment as described in detail elsewhere [49]. Briefly, the sample DNA was diluted to 4 ng/µL in a volume of 40µL, sonicated to an average size of 290 bp using a Q800R2 sonicator (QSonica, Newtown, CT, USA), and a short-read next-generation library was prepared using Agilent Sure-Select methodology. The library was then subjected to two rounds of DNA capture and enrichment and then sequenced on an Illumina MiSeq using a MiSeq Reagent Nano Kit v2 500 cycle kit (2 × 250).

Kidney samples from 18 additional dolphins from the Southern California Bight were submitted for PCR and all were negative. These samples were from long-beaked common dolphins (*Delphinus delphis bairdii;* *n* = 11) and short-beaked common dolphins (*n* = 7) collected between the years 2002—2019.

### Bioinformatic methods

**Fig. 3:**
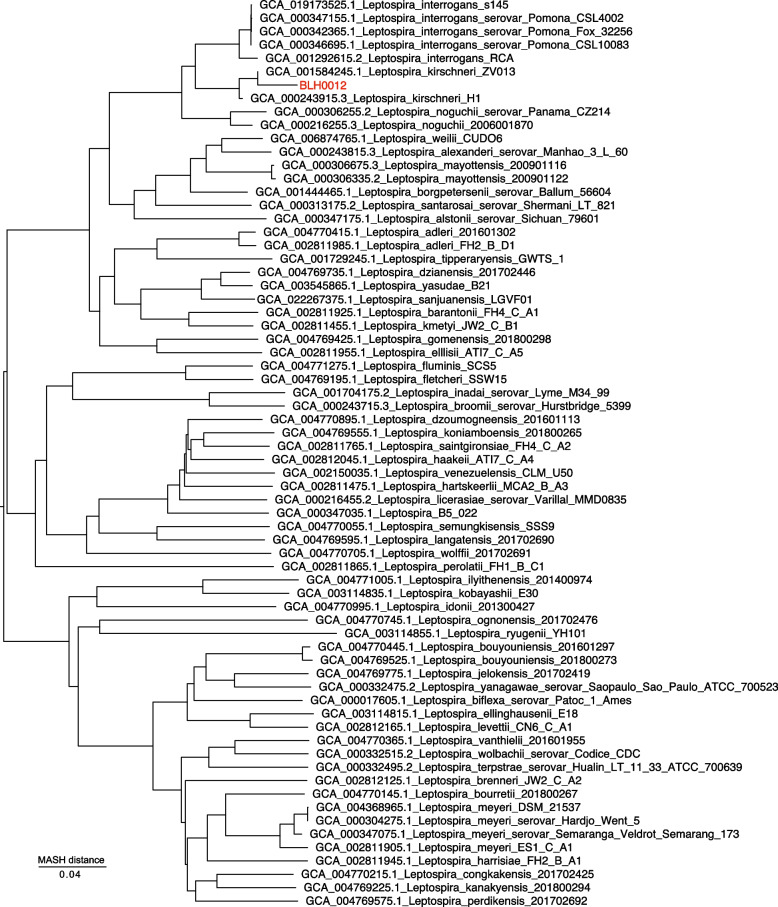
Whole-genome dendrogram of *Leptospira* genomes, showing dolphin sequence BLH0012 within a clade of *L. kirschneri *isolates. The dendrogram includes representative genomes of 63 *Leptospira* species from the P1, P2, S1 and S2 clades. [6], three *L. interrogans* serovar Pomona genomes derived from isolates obtained from two California sea lions and a Channel Island fox (*Urocyon littoralis*), and the enriched *Leptospira* genome assembly from sample BLH0012 (in red)

To estimate the percentage of *Leptospira* reads in the enriched sequences, reads were mapped against the standard Kraken database with Kraken v2.1.2 [50]. Reads assigned as *Leptospira* were then extracted and assembled using SPAdes v3.13.0 [51] with default settings. The BLH0012 assembly was placed into a genus dendrogram with Mashtree v1.2.0 [52] to confirm species membership. The large scale Blast Score Ratio (LS-BSR) tool v1.2.3 was used to identify a set of 131 DNA capture and enrichment probes that had a blast score ratio (BSR) value [53] of ≥ 0.8 in 35 *L.kirschneri* genomes and < 0.4 in other *Leptospira* genomes (*n* = 620). Reads from the short-beaked common dolphin *Leptospira* enriched genome (BLH0012) were mapped against these probes and the breadth of coverage was calculated at 3 × depth. Single nucleotide polymorphisms (SNPs) were identified among the BLH0012 enriched genome and 41 publicly available *L.kirschneri* genomes (GenBank accession numbers annotated in Fig. 3) by aligning reads simulated by ART vMountRainier [54] against *L.kirschneri* serovar Valbuzzi str. 200,702,274 (GCA_000244515.3) with minimap2 v2.22 [55] and calling SNPs from the BAM file with GATK v4.2.2 [56] using a depth of coverage ≥ 5 × and a read proportion of 0.9. All of these methods were wrapped by NASP v1.20 [57]. A maximum likelihood phylogeny was then inferred on the concatenated SNP alignments using IQ-TREE v2.2.0.3 with default parameters [58] (Minh et al. 2020), and the integrated ModelFinder method [59]; the phylogeny was rooted with *L.santarosai* strain LT821 (GenBank assembly accession: GCA_000313175.2). To explore the possibility that the *Leptospira* lineage present in this sample has been described previously but perhaps without genomic level resolution, we queried our assembly against the *Leptospira* pubMLST database [60], which contained 217,925 *Leptospira* allele sequences on the date of access [61].

### Phylogenetic results

Post enrichment, 81.45% of the BLH0012 sequencing reads assigned to *Leptospira* (1,323,517/1,624,860). The whole genome dendrogram, which included representative genomes of 63 *Leptospira* species from the P1, P2, S1 and S2 clades [6], three *L. interrogans* serovar Pomona genomes derived from isolates obtained from California sea lions and a Channel Island fox (*Urocyon littoralis*), and the enriched *Leptospira* genome assembly from the dolphin kidney sample, BLH0012, placed it closest to *L.kirschneri* (Fig. 3). Of 131 likely *L.kirschneri*-specific 120bp RNA capture probes that were included in our enrichment system, 87 were identified in the BLH0012 enrichment with ≥ 3 × coverage, providing further supporting evidence that this unknown *Leptospira* is most similar to *L.kirschneri* and falls within the phylogenetic clade of that species [62].

**Fig. 4:**
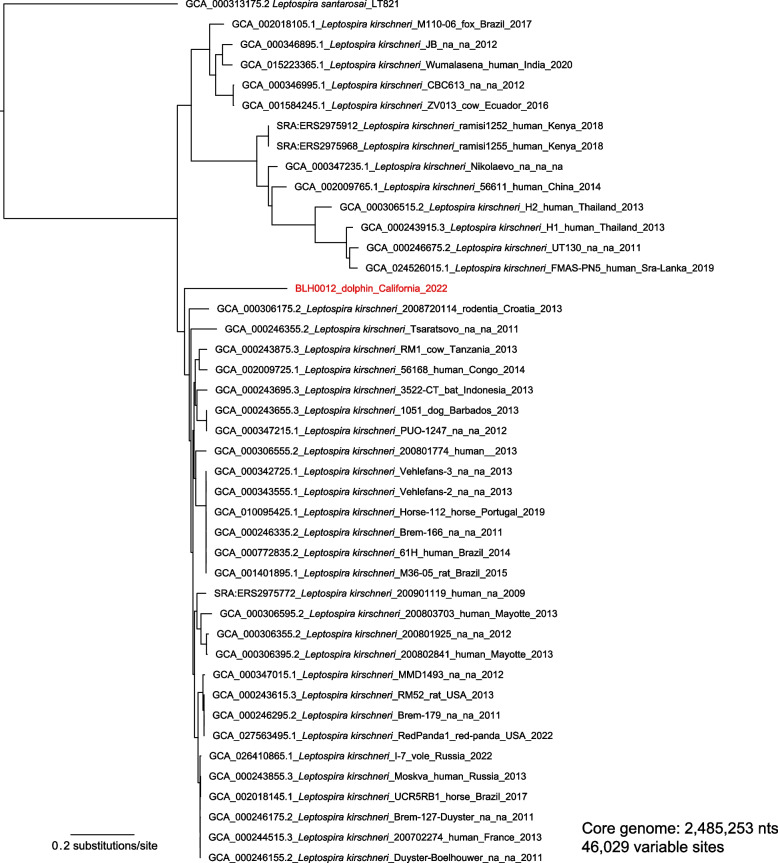
Phylogeny of *L. kirschneri* genomes, showing that the BLH0012 genome represents a divergent lineage of *L. kirschneri*. This whole genome SNP phylogeny included BLH0012 *Leptospira* sequences (in red) and 41 *L. kirschneri* genomes. The dolphin sequence falls among the *L. kirschneri* genomes, with long branch length (7893 unique SNPs) suggesting that it is a divergent lineage

To more definitively assess the relationship of BLH0012 within *L.kirschneri*, we constructed a whole genome SNP phylogeny using the BLH0012 genome and 41 publicly-available *L.kirschneri* genomes previously generated from isolates. This analysis clearly placed the BLH0012 genome among the *L.kirschneri* genomes, confirming the species identification. However, the long branch length leading to the BLH0012 genome (7892 unique SNPs; Fig. 4) suggests it is the first representative of a previously undescribed novel lineage within *L.kirschneri*. In support of this conjecture, our query of the enriched BLH0012 assembly against all *Leptospira* loci in pubMLST revealed the closest allelic matches were to *L.kirschneri* but no perfect matches to any known *Leptospira* alleles at any locus; 6/7 loci were identified from both *Leptospira* MLST scheme 1 (*tpiA* was absent) [63] and scheme 3 (*rrs* was excluded in the bait design) [49, 64], and 7/7 loci were identified from scheme 2 [65]. Overall, our analyses of the *Leptospira* genome enriched from dolphin kidney sample BLH0012 suggest it represents a previously undescribed, divergent and novel lineage of *L.kirschneri*.

## Discussion and conclusions

We report the first detection and characterization of *Leptospira* infection in a short-beaked common dolphin and the first detection of *Leptospira* in any cetacean from the northeastern Pacific Ocean. Renal lesions identified on histopathology of samples from this dolphin were consistent with clinically significant leptospirosis in other host species, including marine mammals [2, 4, 18, 19, 27, 31–34, 66]. These lesions were the most significant lesions detected, suggesting that leptospirosis most likely played a significant role in live stranding and eventual death. We identified the isolate as belonging to a divergent, previously undescribed lineage of *L.kirschneri*, a species that has been detected only once in the northeastern Pacific (or in any marine host): from a single northern elephant seal stranded in northern California in 2004 [31]. Although antibody reactivity against *L.kirschneri* serovar Grippotyphosa has been detected via microscopic agglutination testing (MAT) in a number of marine mammals from the northeastern Pacific, including Pacific harbor seals, California sea lions, and northern elephant seals [32, 33, 36, 37], titers against other *Leptospira* species and serovars were typically also observed in the same individuals. Thus, the MAT cannot be used to definitively identify the infecting species or serovar as *Leptospira* antibody cross-reaction is common [67–69] and not all *Leptospira* species are included in MAT panels. Exposure to unknown species of *Leptospira* has also been detected via MAT in sea otters [66, 70–72].

Infection with *L.kirschneri* distinguishes this case from other observations of *Leptospira* infections in marine mammals of the northeast Pacific Ocean. *L. interrogans* serovar Pomona has caused yearly, seasonal outbreaks in California sea lions for decades, with infections ranging from subclinical to deadly [26, 27, 25, 28–30, 73, 74]. Confirmed infections with *L. interrogans* serovar Pomona have also been detected by culture in northern elephant seals [37] and northern fur seals [34, 35], and by PCR in a Steller sea lion [31]. The different species of *Leptospira* indicates there is no direct connection between these observations of *Leptospira* in marine mammals in the northeastern Pacific, but it is important to note that sampling and testing have been limited. Further *Leptospira* surveillance of marine mammals, including of short-beaked common dolphins, is needed to identify other hosts carrying closely related strains of *L.kirschneri*, which would indicate potential intra- or interspecies transmission linkages and the host range for this novel lineage of *L.kirschneri*. It is also possible that the dolphin was infected by cross-ecosystem spillover transmission from a terrestrial host [75, 76]; *L.kirschneri* has been detected in a range of terrestrial hosts, including rodents, horses, cattle, dogs, humans and wild boar [77–91].

Confirmed infection of a cetacean raises interesting questions about the mechanism of transmission, given the obligate marine lifestyle of cetaceans (in contrast to pinnipeds, which spend time on land) and the commonly accepted view that *Leptospira* are quickly killed by salt water. If there is intraspecies transmission among short-beaked common dolphins, it could be occurring via vertical or sexual transmission, preventing exposure to salt water. In this case, sexual transmission is unlikely given the age of the dolphin; however vertical transmission may have occurred as it was likely still nursing. Expanded surveillance and testing in this species would be needed to assess whether infection prevalence, age distribution, and tissue distribution are consistent with this possibility. If transmission occurred via the environment, whether from another marine host (of the same or a different species) or cross-ecosystem spillover from a terrestrial host, the pathogen would need to survive for some period in sea water. However, *Leptospira* are generally understood to survive poorly in salt water [21, 23, 24]. Some researchers have reported halophilic or halotolerant pathogenic *Leptospira* species; yet in all of these cases isolates were cultured in nutrient rich media of varying salinity which was often less than that of sea water [40, 41, 92]. In addition, work by Trueba et al. [24] suggests that the inhibitory impacts of salinity are most important under starvation conditions (i.e., what would be experienced in the ocean), hence in the absence of nutrient rich media the reported halotolerant and halophilic species are unlikely to survive long. Finally, Saito et al. [93] showed that isolates of the pathogenic species *L.kmetyi* were killed within 12 h in 3% NaCl solution (i.e., the same salinity of the ocean), but were able to survive 3–4 days if incubated with soil overnight. These data, together with considerations of rapid dilution in circulating ocean water, suggest that the window of opportunity for environmental transmission would be quite short for dolphins, and would be more likely in coastal species such as bottlenose dolphins. However, under certain optimal conditions (large aggregations of animals, or behaviors involving particularly close contact with urine) intraspecific environmental transmission might occur in marine settings. This could have implications for understanding *Leptospira* ecology in pinnipeds as well, where transmission has broadly been assumed to occur while animals are hauled out on land.

Ultimately, to better understand the extent to which *Leptospira* infections occur in the marine ecosystem and the epidemiological linkages between and among marine and terrestrial host species, surveillance and sampling must be expanded across these ecosystems. Sequencing of *Leptospira* genomes from different host species will yield crucial information about possible transmission links, either through sequencing of isolates obtained via culture of prospective samples, or by application of DNA enrichment techniques to both banked and prospectively collected samples that test positive by PCR. The advent of these new techniques ushers in a new era for understanding and untangling the complex ecology and transmission of this important zoonotic pathogen.

## Data Availability

Sequence data for sample BLH0012 was deposited under BioProject PRJNA1029025; https://www.ncbi.nlm.nih.gov/bioproject/PRJNA1029025.

## Abbreviations

BSR: Blast score ratio
DNA: Deoxyribonucleic acid
EMJH: Ellinghausen–McCullough–Johnson–Harris
H&E: Hematoxylin and eosin
IHC: Immunohistochemistry
LS-BSR: Large scale BSR
MAT: Microscopic agglutination testing
MLST: Multilocus sequence typing
PCR: Polymerase chain reaction
SNP: Single nucleotide polymorphism
